# The Promise and Challenges of Integrating Biological and Prevention Sciences: A Community-Engaged Model for the Next Generation of Translational Research

**DOI:** 10.1007/s11121-024-01720-8

**Published:** 2024-09-03

**Authors:** Leslie D. Leve, Mariano Kanamori, Kathryn L. Humphreys, Sara R. Jaffee, Robin Nusslock, Veronica Oro, Luke W. Hyde

**Affiliations:** 1 Prevention Science Institute, University of Oregon, Eugene, USA; 2 Department of Counseling Psychology and Human Services, University of Oregon, Eugene, USA; 3 Department of Public Health Sciences, University of Miami Miller School of Medicine, Miami, USA; 4 Department of Psychology and Human Development, Vanderbilt University, Nashville, USA; 5 Department of Psychology, University of Pennsylvania, Philadelphia, USA; 6 Department of Psychology & Institute for Policy Research, Northwestern University, Evanston, USA; 7 Department of Psychology & Survey Research Center at the Institute for Social Research, University of Michigan, Ann Arbor, USA; 8 Cambridge Public Health, University of Cambridge, Cambridge, UK

**Keywords:** Genomics, Neuroimaging, Prevention, Integration, Community-based participatory research

## Abstract

Beginning with the successful sequencing of the human genome two decades ago, the possibility of developing personalized health interventions based on one’s biology has captured the imagination of researchers, medical providers, and individuals seeking health care services. However, the application of a personalized medicine approach to emotional and behavioral health has lagged behind the development of personalized approaches for physical health conditions. There is potential value in developing improved methods for integrating biological science with prevention science to identify risk and protective mechanisms that have biological underpinnings, and then applying that knowledge to inform prevention and intervention services for emotional and behavioral health. This report represents the work of a task force appointed by the Board of the Society for Prevention Research to explore challenges and recommendations for the integration of biological and prevention sciences. We present the state of the science and barriers to progress in integrating the two approaches, followed by recommended strategies that would promote the responsible integration of biological and prevention sciences. Recommendations are grounded in Community-Based Participatory Research approaches, with the goal of centering equity in future research aimed at integrating the two disciplines to ultimately improve the well-being of those who have disproportionately experienced or are at risk for experiencing emotional and behavioral problems.

Personalized health interventions based on one’s biology are on the rise. Although advances have been made in personalized medicine approaches for disease conditions such as cancer (Simona et al., 2023; Singh, 2023), there has been limited progress on the implementation of personalized approaches for emotional and behavioral health problems such as depression, substance use disorders, or antisocial behavior. At the same time, the availability and impact of interventions aimed at preventing emotional and behavioral health problems have never been higher, as reflected in the number of emotional and behavioral prevention programs meeting criteria for being “evidence-based” on national registries. For example, more than 100 programs are listed on one national resource—Blueprints for Healthy Youth Development—as “promising,” “model,” or “model plus” interventions in terms of the rigor of their evidence base (https://www.blueprintsprograms.org/program-search/). The purpose of this paper is to describe advances in biological science approaches, with a focus on genomics and neuroimaging, and to describe how these might inform current directions in the field of prevention science. To this end, the Society for Prevention Research convened a multidisciplinary task force charged with addressing the integration of biological and prevention sciences, one outcome of which is this manuscript.

We begin this manuscript by looking back to the turn of this century (2000), when biological methods and technologies were advancing rapidly in many domains. We describe new approaches for collecting and analyzing human DNA samples to understand genetic influences on behavior that were increasingly accessible to researchers, and in parallel, advances in neuroimaging methods that provided a new window into the brain. During this time, early studies that integrated biological science and prevention science approaches began to appear in scientific journals, including *Prevention Science* (e.g., Bruce et al., 2009; Sales et al., 2014). As the science advanced, so too did recommendations for the successful integration of these methods within prevention science (for additional reading related to addressing challenges linking research to practice and policy in prevention science, see Crowley et al., 2018; Fishbein, 2000, 2016; Fishbein & Dariotis, 2019; Fishbein et al., 2016).

Despite the excitement generated by these early studies and papers, efforts to integrate biological sciences with prevention science have faced a number of challenges. We review these challenges in the next section of this manuscript, and we explain why we suggest that biological science has not fully realized its promise of transforming prevention science to inform personalized health interventions. We highlight complexities in the science that pose real hurdles for true integration of the two disciplines while discussing the lack of diversity in participants and researchers, the need for collaboration with community partners, challenges in the interpretation of the data, and ethical considerations. This presents a realistic, albeit somewhat pessimistic, outlook on the barriers that must be overcome in order for the two disciplines to become integrated in a way that equitably advances science to improve human health and well-being using personalized approaches.

Our skepticism turns to optimism, however, as we enumerate specific approaches that we believe would allow for better, more equitable, and responsible integration of biological science into prevention science research and practice. This includes grounding such work in a Community-Based Participatory Research (CBPR) model; forming meaningful collaborations between community members, experts in biological science, and experts in prevention science; developing and deploying improved analytical approaches; committing to professional development-oriented conversations around racism (and other structural inequities) and biomarker science before embarking on such collaborations; and including transdisciplinary experts on grant and editorial board review panels. We follow this section with a description of proposed steps to apply a CBPR framework to research investigations that include prevention science and biological science methods, noting the benefits and challenges to communities and researchers in each step in the process. In the penultimate section of the manuscript, we provide several examples from the field of prevention science that have made advances in one or more of the areas of integration described in the prior section. We conclude with a discussion of ongoing barriers, future areas of opportunity, and recommendations.

## Defining What We Mean by “Biological Science” in This Report

This paper focuses on two biological science methods advances of the twenty-first century: genomics and neuroimaging. We refer to these as “biomarkers” throughout this report, to indicate a biological characteristic that reflects variation in processes or mechanisms that can be objectively measured, such as a gene sequence from analysis of a person’s DNA or a measure of gray matter volume from a scan of a person’s brain. We acknowledge that there are many other approaches that can directly assay biology, such as electroencephalography (EEG), cortisol collections via hair or saliva, or measures of the immune system (e.g., Nusslock & Miller, 2016). These are not used as examples within this report, due to space considerations. Similarly, there are advances in genetically informed research designs (e.g., children of twins studies) that may be relevant to prevention science, but are not detailed in this report. Nonetheless, many sections of this manuscript could apply broadly across a range of biological science methods, and we encourage readers to consider the challenges and recommendations described in this manuscript with a view toward the specific biological science method(s) that they are using or plan to incorporate.

### Advances in Genomics: DNA Sequencing, Genome-Wide Association Studies, Polygenic Score Computation, and Epigenetics

The completion of the Human Genome Project in 2003, which identified the DNA sequence (i.e., the sequence of nucleotides) of the entire human genome (Green et al., 2015), generated tremendous excitement about the possibility that knowledge of a person’s DNA would provide information about their disease risk and their treatment response. Soon thereafter, the first genome-wide association study (GWAS) was published (Klein et al., 2005). Although approximately 99.9% of the genome is identical from one person to the next, the 0.1% that is not shared represents three million genetic variants and all their combinations, giving rise to a wide range of individual differences in behavior, cognition, and risk for disease. This variation is captured in GWAS. Unlike candidate gene studies, which measured variation in a single gene at a time and have fallen out of favor due to replication failure (Duncan et al., 2019), GWAS measure hundreds of thousands to millions of gene variants (called single-nucleotide polymorphisms, or SNPs) across the genome. Statistical geneticists use GWAS data to calculate polygenic scores, which are weighted combinations of gene variants that, additively, account for meaningful proportions of variance in phenotypes of interest and have been used with modest success in individual risk prediction models (Murray et al., 2021). An advantage of this method is that scores can be transported to smaller, deeply phenotyped samples, thus enabling researchers to test hypotheses about gene-environment interaction or correlation. However, a downside of these polygenic scores is that they may provide little insight into the underlying mechanisms that may be driving the outcomes to which they are linked, undermining their translational value (Visscher et al., 2021). Thus, increased predictive power has outpaced biological insight. Regardless, polygenic scores derived from these GWAS are being used in clinical risk prediction models to improve our ability to predict and prevent disease.

In addition, researchers are attempting to model interactions between biological processes and environmental experiences by measuring epigenetic modifications or gene expression (Jones et al., 2018; Miller et al., 2009). Epigenetic marks, such as DNA methylation, are modifications to the packaging of DNA that can influence whether a given gene will be expressed, or “turned on.” In contrast to the sequence of DNA, which is set at conception and for the most part is static across the lifespan, epigenetic markers and levels of gene expression can undergo dramatic changes over the course of development and in response to environmental exposures and life experiences (Jones et al., 2018). There are several reasons why it is challenging for researchers to determine whether epigenetic modifications and changes in gene expression play a causal role in the etiology or maintenance of emotional and behavioral health problems (Walton et al., 2019), including the lack of studies with repeated assessments of both DNA methylation and measures of emotional and behavioral health problems, the use of peripheral tissue (which can be sampled from live humans) instead of brain tissue (which cannot currently be sampled from live humans), and lack of ability to isolate epigenetic effects from other potential mechanisms (e.g., epigenetic patterns are heritable).

Despite these limitations, these advances in DNA assay and statistical analysis approaches have begun to be incorporated into prevention science research and present opportunities for the integration of biomarkers into prevention science studies (Li et al., 2022; Neale et al., 2021).

### Advances in Neuroimaging

The 1990s was termed the “Decade of the Brain,” and, in part due to the initiation of the Human Genome Project (1990–2003), sparked an explosion of interest in linking health and illness to brain structure and function (Jones & Mendell, 1999). This interest was fueled, in part, by the development of magnetic resonance imaging (MRI). Structural MRI uses magnetic gradients and electromagnetic fields to generate high-resolution images of biological tissue. Researchers can use structural MRI to examine relationships between the volume, thickness, or surface area of a particular brain region (or connections between areas when using diffusion imaging) with clinical outcomes or other behavioral and psychological variables. Studies involving structural MRI have reported that many mental health problems, including emotional and behavioral disorders, are characterized by individual differences in the structure of brain regions that generate and regulate emotions (e.g., Shackman et al., 2016; Treadway, 2016). In some cases, these structural brain differences pre-date the onset of the observed illness (Borgwardt et al., 2007; Foland-Ross et al., 2015), suggesting they may indicate pre-existing risk factors and can help identify individuals for possible preventive interventions (Rashid & Calhoun, 2020). Information obtained via structural MRI is limited, however, by its static representation of tissue. Functional MRI (fMRI) complements structural imaging by generating maps of possible neuronal activation that can be linked to more dynamic mental processes. Using fMRI, researchers can measure changes in brain function while participants perform experimental tasks, or at rest, and then relate these changes to clinical, behavioral, and/or psychological variables (Heeger & Ress, 2002). As with the advances in genomic methods, these new tools provided researchers access to data about the brain that was unavailable just a few decades ago and offer the potential for the integration of prevention science and neuroimaging approaches to inform precision medicine approaches.

### The Promise of Translation

Consistent with a precision medicine approach, a goal of increased emphasis on biological risk factors and mechanisms has been to identify: (a) mechanisms through which mental and physical health problems emerge, (b) individuals who may be at higher risk (to be targeted via prevention), and (c) subgroups of individuals who may have similar symptoms, but distinct causes to their health challenges (e.g., Gratton et al., 2019; Insel & Cuthbert, 2015). If biomarkers can help to identify these mechanisms, risk factors, and subgroups, they could lead to early identification of individuals for prevention purposes, targeted and personalized interventions for individuals with different causes, and/or new prevention and intervention targets via better understanding of the causes and mechanisms of health and illness (Hyde, 2015). Thus, at the broadest level, biomarker approaches offer new inroads for prevention science by offering new ideas on who to target, how to target, and what to target in prevention and intervention efforts. In addition, at the individual level, biological mechanisms may partially explain prevention effects and serve as putative mediators. Including biomarkers in the context of a prevention trial has the potential to inform our understanding of why prevention studies typically have small to modest effect sizes, and to help explain the heterogeneity in intervention outcomes. This information could then be used to guide refinements to existing prevention programs or to guide the development of new programs that focus on novel targets, with the potential to benefit more people when applied in the context of interventions with at least modest effect sizes, strong implementation, and high levels of participant engagement (e.g., Leve et al., 2010). These directions are discussed further in the penultimate section of this manuscript.

**Fig. 1:**
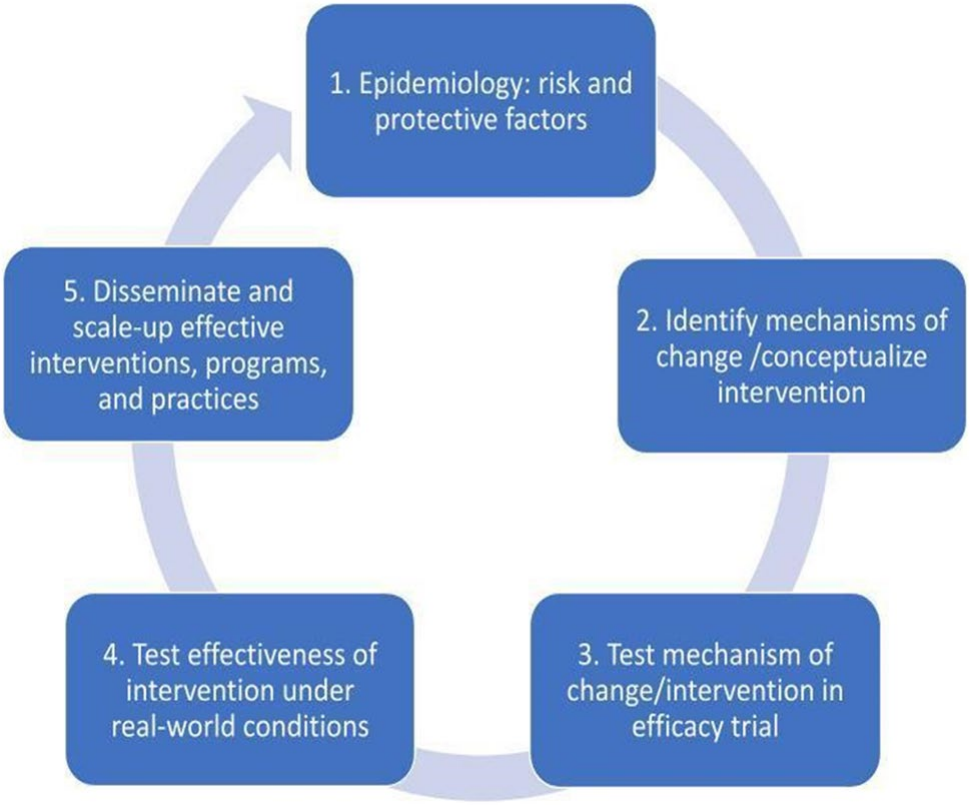
Prevention Research Cycle. Note: Adapted from the Institute of Medicine (1994)’s five-step model for assessment, intervention, and dissemination

This precision medicine conceptualization of the utility of biomarkers for advancing the understanding of emotional and behavioral health problems is synergistic with the broad definition of prevention science as having a primary goal of improving public health by “identifying malleable risk and protective factors, assessing the efficacy and effectiveness of preventive interventions, and identifying optimal means for dissemination and diffusion” (Biglan et al., 2011). In particular, within the prevention research cycle (illustrated in Fig. 1, adapted from the Institute of Medicine, 1994), phase #1 includes conducting research to understand predictors of problem and positive developmental outcomes and understanding the epidemiology and natural history of the problem, phase #2 includes developing interventions to motivate changes in individuals, groups, and environments based on theories of human behavior and our understanding or mechanisms for behavior change, and phase #3 includes testing the efficacy of these preventive interventions and their mechanisms under tightly-controlled parameters and settings (Biglan et al., 2011; Fishbein, 2016). Examples of biomarker science that has been conducted within phases #1–3 of the prevention research cycle are presented in a later section of this report. Phases #4–5 involve testing effectiveness in real-world settings and dissemination efforts and are not a focus of this report because biomarkers have not generally been used in these phases.

### Where is the Field Today? Challenges in Advancing Integrated Biomarker-Prevention Science Research

Despite broad enthusiasm for integrating biological and prevention sciences to inform precision medicine approaches, implementing this vision has been challenging. We discuss four sets of challenges that have impeded progress in this section, before turning to strategies to address these challenges in subsequent sections.

#### The Complexity of the Science Exceeded Initial Expectations

A major challenge in advancing an integrated biological sciences-prevention science agenda is that linking biomarkers to emotional and behavioral health outcomes has been more complicated than initially expected. When the Human Genome Project was completed, researchers began looking for gene variants that underlie mental health problems. The hope was that a small number of gene variants would explain a large amount of variance in psychopathology outcomes, similar to genetic mutations like the *BRCA1* and *BRCA2* mutations implicated in breast and ovarian cancer. Initial efforts were also informed by experimental animal models, including mouse knockout and behavioral neuroscience studies that showed how behavior was affected when specific genes or neurotransmitter systems were effectively silenced (Cases et al., 1995; Murphy et al., 2001; Shih & Thompson, 1999). As a result, researchers initially focused their efforts on variants in genes such as the serotonin transporter (*5-HTTLPR*), monoamine oxidase A (*MAOA*), dopamine receptor 4 (*DRD4*), and dopamine transporter (*DAT*) genes that were known to be associated with risk for various psychiatric problems, including depression, substance use disorder, and attention-deficit/hyperactivity disorder (ADHD; Caspi et al., 2003; Okuyama et al., 2000; Rowe et al., 1998). Yet, these single gene variants only accounted for a very small percentage of variance in emotional and behavioral health outcomes (2–4%), and frequently did not replicate in different cohorts (Risch et al., 2009) or in well-powered GWAS (e.g., Farrell et al., 2015; Flint & Munafò, 2013). Gradually, however, it became clear that individual differences in complex human traits were explained by hundreds or thousands of gene variants, each contributing a very small percentage of risk, and not by a small number of genes of large effect (Visscher et al., 2021). Moreover, as the science evolved, it has become apparent that the genetics of emotional and behavioral health (and many forms of physical health) do not follow a simple Mendelian pattern in which one gene is associated with one outcome. Polygenic scores that increase risk for one psychiatric disorder are usually associated with other disorders as well, and their effects may be contingent on both the expression of one or more independently inherited genes as well as the environment (Lee et al., 2019, 2021; Smoller et al., 2019). Even with large ensembles of genetic variation measured, the cumulative effects of these genes still only explain a relatively small percentage (< 10%) of variation in behavior (Gibson, 2010), highlighting the complexity of ways in which multiple genetic variants, combined with specific environmental exposures, likely influence emotional and behavioral health outcomes. It is possible that with new, theoretically driven multivariate gene identification methods, polygenic scores will begin to account for as much variance in emotional and behavioral health outcomes as some of the more robust social risk factors, such as socioeconomic status (see Karlsson Linnér et al., 2021, for more information on such approaches).

Unfortunately, things are not simpler in the brain, guided by neuroimaging research advances. Historically, structural and functional neuroimaging studies examined brain regions in isolation of each other. Although this approach was intuitively appealing and offered more direct explanatory power, simple findings linking the structure or function of a single brain region to emotional and behavioral health outcomes have not been replicated, nor shown consistent predictive power (Botvinik-Nezer & Wager, 2023). There is growing recognition now that emotional and behavioral health and illness may be driven by complex connections among these brain regions, rather than the size or activity in a single region (Bassett et al., 2018), and important advancements are being made in determining the best methods for characterizing such networks (Barack & Krakauer, 2021; Basset et al., 2018; Bassett et al., 2018). Further, and similar to genomics, individual differences in one brain region or circuit may be implicated in numerous different emotional and behavioral health conditions, reflecting transdiagnostic, rather than specific, biomarkers of risk (Insel & Cutherbert, 2015). Moreover, as with genomics, as neuroimaging identifies more and more complex brain patterns to be associated with outcomes, it is becoming clear that the effects are relatively small, thus requiring very large samples to identify relatively nuanced connections between brain and behavior (Feng et al., 2022; Marek et al., 2022). This complexity has made it difficult to identify biomarkers that can reliably identify vulnerable individuals and differentiate individuals at risk for one emotional or behavioral health problem from another. This complexity has also made it difficult to identify translational biological processes that can be targeted in prevention or intervention programs.

#### Lack of Diversity

A second major barrier to progress is a lack of diversity in existing biomarker science, including concerns regarding: (1) who is the focus of the research, (2) who is conducting the research, and (3) how is community involvement integrated into the research. These concerns are not unique to biomarker science and permeate other translational disciplinary efforts as well; challenges related to the integration with prevention science are described below.

##### Lack of Diversity of Participants

Typically, partially due to cost, many biomarker studies, particularly human genomics, and neuroimaging studies, are conducted with convenience samples, often of relatively socioeconomically advantaged, primarily White/European individuals residing near major universities (e.g., a bias toward those living in suburban and urban settings versus those living in rural areas; Falk et al., 2013). This is an extension of the broad issue in social sciences of focusing on WEIRD (Western, Educated, Industrialized, Rich, and Democratic) populations (Henrich et al., 2010). Although some recent large-scale studies are leading to improvements in sampling (e.g., the Adolescent Brain Cognitive Development [ABCD] Study [Hagler et al., 2019]; neuroimaging with the Future of Families and Child Wellbeing Study [Goetschius et al., 2020]; the Environmental influences on Child Health Outcomes [ECHO] study [Knapp et al., 2023]), it is not clear from most published research to date who the research generalizes to because of the use of convenience sampling (Falk et al., 2013). Beyond the philosophical issue of generalizability, the field has also failed to include participants from diverse socioeconomic, ethnic, and racial groups, and studies often do not even report the demographics of participants (Qu et al., 2021). This poses challenges for translation to prevention science, given that focal populations for prevention efforts are often from marginalized and/or underrepresented groups. What if associations between a specific biomarker and a measure of emotional or behavioral health differ between the extant literature and the population of focus in the prevention study? Racial and ethnic minorities are underrepresented broadly in biomedical research. White/European Americans make up 67% of the U.S. population, but are 83% of research participants (Taylor, 2019; Yates et al., 2020). Black/African Americans make up 13% of the U.S. population, but only 5% of participants, and Hispanic and/or Latino/a/x/e (hereafter referred to as Latine) represent 18% of the U.S. population, but less than 1% of participants (Yates et al., 2020).

Human genetics research faces the challenge that historically, GWAS have not represented population-wide genetic diversity. Over hundreds of thousands of years, different groups of people had different patterns of migration, adapted to different environments, and had different patterns of mutations and recombination, leading to distinct genetic signatures, reflected in patterns of linkage disequilibrium and allele frequencies. These genetic ancestry patterns are statistically correlated with social categories of race and ethnicity but are not identical. For example, genetic diversity is greater on the African continent than in the rest of the world combined, but most of this diversity has not been sampled (Choudhury et al., 2020). Thus, polygenic risk scores derived from individuals of European genetic ancestry do not capture the genetic variation present in individuals of, for example, African genetic ancestry and, as a result, are not as predictive of health outcomes (or any other phenotype) when applied to other ancestral groups (e.g., Duncan et al., 2019; Mars et al., 2022). Thus, there is the possibility that the use of these polygenic scores in personalized medicine from GWAS of individuals of European ancestry will exacerbate existing health disparities related to race and ethnicity (Martin et al., 2022), when considered in the context or a prevention trial. In recognition of this problem, there are new initiatives to increase the representation of diverse groups in GWAS (e.g., BioBank Japan, the Latin American Genetics Consortium, H3Africa Consortium, NIH’s All of Us Research Program), new platforms for genotyping DNA from diverse groups, and new methods for analyzing GWAS data across ancestral groups and within groups of mixed genetic ancestry (e.g., Hispanic and/or Latine participants; Atkinson et al., 2021; Peterson et al., 2019). Recent reports are already showing that increasing ancestral and global diversity in genetic studies can help increase the discovery of core genes and increase the transferability of findings (Meng et al., 2024).

It is likely that the representation of marginalized communities is even lower in neuroimaging studies (Gard et al., 2020; Qu et al., 2021). This lack of representation is problematic as it undermines our understanding of “the human brain” and how variations in brain structure and function are impacted by experience and predict health outcomes. If biomarker studies do help to identify biological mechanisms that could potentially be changed through intervention (prevention research cycle phase #2), or test ways to personalize prevention based on a biological characteristic (prevention research cycle phase #3), but this science is based on a small, homogeneous subset of the population, then disparities in positive outcomes from prevention and intervention programs will increase. That is, the lack of proportional representation could potentially lead to interventions that are not efficacious in other populations (Yates et al., 2020), are not effective for many populations (Bass, 2020), or do not translate well into real-world use (Yates et al., 2020).

##### Lack of Diversity of Researchers

Issues of inclusion and generalizability in genomics and neuroimaging study samples may be partially related to lack of diversity among those leading the research, both in their identities and in their training and expertise. Although we are unaware of an analysis that identifies the demographics of genomics researchers, people with racial and ethnic identities that are marginalized are under-represented in adjacent fields (e.g., psychology; Hur et al., 2017) and broadly in biomedical research (Ricard et al., 2022). Moreover, recent work has shown that, within neuroscience, White authors tend to disproportionately cite other White authors (Bertolero et al., 2020), and faculty with marginalized identities receive less federal funding than White faculty (Hoppe et al., 2019). Thus, our field underrepresents many racial and ethnic identities and there are clear barriers to success in the field for those with marginalized identities. Increasing researcher diversity is likely to broaden the range of questions researchers consider relevant, increase the ease with which researchers engage with participants from marginalized communities, and expose hidden biases in the interpretation of findings from biomarker research (Rowley & Camacho, 2015).

Beyond the lack of racial and ethnic diversity of researchers engaging in biomarker research and prevention science research, an additional challenge is the need to bridge sources of knowledge to ensure the multidisciplinarity of research on integrated biomarker-prevention science research. Many prevention scientists recognize that biological risk factors interact in complex ways with each other and with other non-biological risk factors (e.g., Fishbein, 2000). This recognition can lead prevention scientists to collect multiple forms of biological, social, and cognitive data in a desire to model this complexity. However, none of us can be experts in everything, and a challenge with successful interdisciplinary biomarker-prevention science research is forging collaborations that bring together the requisite expertise to elevate the research beyond the sum of its parts. These collaborations often take time to establish and require researchers from different disciplinary backgrounds to establish common frameworks for defining key constructs and for thinking about key questions. Preferred publication outlets may also differ between the disciplines, as well as expectations around timelines and authorship roles. Moreover, individuals from different disciplines may value or have concerns about different types of approaches (e.g., community-based researchers may have concerns about biological approaches, biologically focused researchers may not see added value in community-based research). To promote successful interdisciplinary partnerships, a coordinated plan for collaboration and dissemination of the science must be established early in the research process.

A lack of interdisciplinary diversity, challenges with collaborating across disciplines (e.g., genomics, neuroscience, prevention science, community-engaged research, public health), challenges in obtaining training across these areas, and a lack of diversity among investigators has likely undermined the extent to which biomarker research has engaged directly with marginalized communities, underrepresenting those who might be more likely to benefit from selective preventive intervention studies and/or individuals who have been marginalized and oppressed in multiple ways (Gilpin & Taffe, 2021). This gap in the translational collaboration pipeline intersects with the lack of diversity among biomarker study participants, limiting the ultimate potential for equitable translation.

##### Lack of Community Engagement

Adding to the aforementioned challenges is the fact that a lack of community engagement can perpetuate mistrust from marginalized communities. Without a history of positive collaboration with communities that may eventually engage with prevention or intervention efforts, biomarker researchers miss individual representation, but also their input. That is, studies involving genomics and neuroimaging rarely include representatives from the participant and community sides, and thus rarely take a community- or participant-focused approach. This unidirectional method of research can lead to interpretation biases (Tolwinski, 2019) and/or scientific directions that do not meet the interests or needs of the community. One example of this is when researchers collected blood samples from Havasupai Tribe members in Arizona to identify a genetic link to diabetes, but later used the samples without the consultation, input, or consent of tribal members, to study genetic linkages with other medical disorders, such as schizophrenia and alcoholism (Sterling et al., 2011). The broader scientific community is beginning to understand the harmful impacts of failure to engage the community in research, as evidenced by the prioritization of patient and stakeholder engagement in some funding priorities (e.g., https://www.pcori.org/engagement/value-engagement) and changes in consenting processes to include “broad consent” if the samples are going to be banked and used for future research.

Just as a lack of community and participant input can undermine the translational value of biomarker research, so too can the lack of collaboration with implementation scientists. Incredible amounts of research and funding are committed to biomarker research with the hope that this basic science can lead to important translation efforts later. Without thinking through how findings could be translated, research efforts may have low translational impact. For example, many of the current directions in biomarker research (e.g., large-scale polygenic scores, complex connectome brain imaging) may not be scalable, nor provide insights at the individual level that are relevant to prevention science. Moreover, many of these approaches are incredibly expensive or inaccessible if participants do not live near a major medical center or research university, leading to the question of whether a typical preventative intervention could have the funding, accessibility, and expertise needed to engage in the real world. That is, how could genome-wide or brain-wide methods be useful in a clinic or the community? These tools are incredibly costly; if a major site for prevention work is community health centers and on-the-ground providers of prevention services, what is the likelihood that these tools can realistically be used at scale in prevention efforts? Thus, it seems unlikely that tools like MRI will be used at scale in prevention work in community settings and thus, it is critical for biomarker researchers to be clear in their work about how it could inform translational goals.

Of course, the advantages that biomarkers promise for greater precision-based intervention may not lay within the use to these tools in community care settings, but rather, in their ability to identify underlying mechanisms that explain variation in responsivity across subtypes of individuals from a range of racial/ethnic groups. Using a neuroscience-informed framework, distinct neurocognitive trajectories that have been recognized as precursors to emotional and behavioral health outcomes could be targeted, and the change processes could be evaluated to inform causal hypotheses. This framework could also inform individualized assessments, intervention development, and outcome measurement in preventive interventions. If successful, the classification and diagnosis that guides prevention and intervention would not be based solely on surveys or interviews, but on sensitive tasks and stimuli previously used during biomarker testing and shown to consistently recruit regions or processes of interest (e.g., neurocognitive tasks, emotion processing indicators, and stress responses) that help us to better understand the key elements and neural mediators of different prevention programs, which could, in turn, help to personalize prevention and intervention and make it more successful. At the same time, these approaches would need to be scaled up to have a broader impact, which is a challenge. Moreover, even in work where the goal is using biological science in empirical studies as a bridge to new and better prevention strategies, an issue remains that if biomarker studies are not done with communities that will be targeted eventually for a prevention trial, then generalizing the results to improve prevention may be challenging and will not reduce health inequities.

#### Challenges in Interpretation

A third challenge in prior biomarker research is related to challenges in interpreting the findings, specifically, concerns regarding sample sizes and measurement.

##### Sample Sizes and Effect Sizes

Early failures with candidate genes (and more recently, with region of interest, task-based neuroimaging studies) and the small effect sizes that have resulted from studies using biomarkers to predict emotional or behavioral health outcomes have led to an acknowledgement of the increased statistical power needed to conduct rigorous and replicable research in this area (Duncan et al., 2019; Marek et al., 2022; Poldrack et al., 2017). This can be a challenge within prevention science, as the costs of implementing interventions often preclude the use of very large samples (particularly in effectiveness trials, within prevention research cycle phase #4 activities). Increasingly, data sharing consortia (e.g., Early Genetics and Lifecourse Epidemiology [EAGLE] Consortium; Middeldorp et al., 2019) and multi-site coordinated data collection efforts (e.g., the ABCD study [Volkow et al., 2018]; HEALthy Brain and Child Development Study [Volkow et al., 2021]; the Environmental influences on Child Health Outcomes study [Knapp et al., 2023]; the All of Us program [All of Us Research Program Investigators, 2019]) are designed to include biological measures as well as measures of experiences and exposures. Unlike the much smaller genomics and neuroimaging studies that were, until recently, common, these large-scale data collection efforts are better-powered to detect the small gene- and brain-behavior associations that appear to be typical and are better powered to detect gene-by-environment interactions. Thus, the field is shifting rapidly, and one of these shifts involves moving to larger consortium studies. This results in a benefit of larger sample sizes and adequate power to detect associations, as well as public data that may be accessed more broadly be a wider variety of researchers, with the potential for more diversity in viewpoints. At the same time, with fewer (but larger) projects, there is also a danger that this process can concentrate the researchers leading this science into a smaller subset of individuals, which can limit innovation and diversity of ideas and scholars—amplifying a threat identified earlier in this section regarding lack of diversity in researchers.

##### Measurement Issues

For biomarkers to be effectively integrated into prevention science studies, researchers must be able to measure them reliably, they must have predictive validity, and there should be some understanding of the pathway from the biological marker or process to the behavior. This can be challenging if, for example, a prevention trial is focused on an underserved community for whom biomarker research has been rare and less likely to have been validated previously. In genomics and neuroimaging research, researchers are still striving to meet these criteria. For example, the acquisition and processing of neuroimaging data (and branching forking of analysis options) creates concerns about reproducibility (for discussion, see Poldrack et al., 2017). In epigenetics research, expert users debate which tissues to sample (e.g., blood, buccal, saliva, hair) and assays to use (e.g., Southern Blot vs. qPCR; https://trn.tulane.edu/), quite apart from the question of whether epigenetic changes in peripheral tissue (versus brain tissue) play any causal role in affect, behavior, or cognition. Even for DNA biomarkers that can be measured reliably, the mapping between biology and behavior is likely complex (i.e., not 1:1) and moderated by developmental and environmental factors (e.g., Tucker-Drob et al., 2013).

#### Ethical Considerations

A final set of challenges discussed in this report, which is one that permeates each of the aforementioned challenges, is that biomarker research broadly, but also specifically in a translational context with goals to inform prevention, can raise potential moral and ethical considerations. First, the eugenics movement, which is a scientifically inaccurate theory that humans can be improved through selective breeding of populations, caused widespread harm beginning in the early twentieth century, particularly to marginalized populations. Some researchers involved in this movement provided inaccurate genetic and/or brain-based justification for these horrific beliefs, leading to a long history of concerns about the use of biological and especially genetic and brain-based measures among many scholars and communities. The eugenics movement has understandably impacted perceptions of the utility of any research that incorporates biomarker data, such as genomics or neuroimaging. It contributed to social disparities that continue into the present in education, medicine, and prevention science, impacting participants’ interest in and acceptance of biomarker research (Prather et al., 2018; Sanchez-Rivera, 2020; Selden, 2000; Winston et al., 2020).

Second, there are valid concerns about confidentiality with biomarker data, particularly the use of DNA data. Violations of privacy and confidentiality in the use of DNA could, theoretically, impact later insurance coverage and/or access to treatments. Even if such a situation never arises, the sheer possibility of a privacy violation may undermine trust between researchers using biomarkers, prevention scientists, and potential participants in a prevention science study.

Third, the use of biomarkers such as genomics and neuroimaging in prevention research can alienate key community leaders and partners, and, combined with the eugenics movement discussed above, has a history of such alienation. The Syphilis Study at Tuskegee, in which Black male participants with syphilis in the study were not offered medical treatments known to be effective in treating syphilis, is one such example. Historical contexts such as this one may alienate participants and community stakeholders who are skeptical of how the data will be used and/or how it will be interpreted (Ricard et al., 2022). This history may also alienate key potential future collaborators (e.g., sociologists) who have important perspectives to share. This justified skepticism contributes to a vicious cycle in which researchers lack key collaborators and community partners, and thus may lack input to make the research more ethical and equitable, which in turn may further motivate those from marginalized and oppressed groups to avoid engaging in integrated biomarker-prevention science research.

Fourth, given many people’s inaccurate intuitions of genes and brain as “immutable,” “in-born,” and “static” (Dar-Nimrod & Heine, 2011), identifying risk biomarkers may lead to stigma and/or self-fulfilling prophecies. The stigma attached for a “risk” biomarker may undermine its use in informing preventative interventions. For example, in one study, participants were randomly assigned to be told that there was either a very high or very low chance that they had a genetic risk for obesity. When asked to select a meal from a menu of options, participants who were told that they were *not* genetically predisposed to obesity were more likely to select unhealthy foods, indicating that personalized feedback that one’s genetic risk is low may increase the likelihood of unhealthy choices (Ahn & Lebowitz, 2018). Fortunately, research has shown that beliefs regarding associations between genes and health outcomes can be changed via brief informational interventions (e.g., Driver et al., 2022).

Fifth, biomarker research may be challenging to interpret, which can lead the public to misinterpret research headlines. For example, a growing number of studies have documented the association between poverty and brain structure and function (e.g., Troller-Renfree et al., 2022). These studies may motivate policy to ameliorate or address the negative impacts of poverty, likely because biological research is viewed as more compelling (e.g., poverty must be “bad” if it impacts children’s brains). It may also help motivate researchers to study this topic and see the potential upside of this research (and how it can inform policy to prevent child poverty). However, this same research may be communicated to the public and to relevant communities (e.g., those with lower income) in ways that increase stigma and undermine potential partnerships with communities at risk. For example, this same research could be incorrectly interpreted by youth as meaning that “poor kids have holes in their brains”—leading to self-fulfilling prophecies and stigma (Tolwinski, 2019). Although researchers understand that correlation does not equal causation, the general public may mistakenly make the false assumption that, for example, people with marginalized identities have mental illnesses like schizophrenia at higher rates due to their genetics, when the science is much more complex than this, given the non-random assortment of people to environments. Thus, there is a clear need for science education in society and amongst clinicians to address some outdated and inaccurate views about biology being static and unmalleable.

These are just some of the complex ethical challenges that must be further acknowledged, discussed, and integrated into the design of prevention science research endeavors before prevention-biomarker science can evolve in an equitable and meaningful manner. If these issues are not discussed, up-front, in potential collaborations and projects, key community partners may be alienated, undermining the success of integrating these fields.

## Strategies to Promote the Responsible Integration of Biological and Prevention Science

Despite the limitations and challenges noted in the prior section, we believe that there are some approaches that could be implemented in the short term to move the field toward more equitable and responsible integration of biological and prevention science. Building on directions led by others in this area (e.g., Dick et al., 2017; Tindana et al., 2015), we present five such approaches in this section, at the center of which is to apply CBPR approaches.

### Adopt a CBPR Approach

As noted in the prior section, racial and ethnic minorities are underrepresented in biomarker research. To increase participation from marginalized communities, prevention science research that involves biomarkers would benefit from leveraging a CBPR approach where community involvement is integrated in all aspects of the research (or even, through the use of some CBPR-consistent approaches to engage with the community). This approach requires the development of mutual trust and bidirectional communication between biomarker scientists, prevention scientists, and communities (Fregonese, 2018), and we believe that it would ultimately help researchers achieve higher-quality research and benefit our society in a more representative manner.

As described by the National Institute on Minority Health and Health Disparities (NIMHD), CBPR “begins with the involvement of and a research topic of importance to the community and combines knowledge with action to improve health outcomes and eliminate health disparities” (https://www.nimhd.nih.gov/programs/extramural/community-based-participatory.html). It is a partnership that equitably involves community members, organizational representatives, and academic researchers in all aspects of the research process. It enables all partners to contribute their expertise, with shared responsibility and ownership; it enhances the understanding of a given phenomenon; and it integrates the knowledge gained with action to improve the health and well-being of community members, such as through interventions and policy change (Israel et al., 1998).

A CBPR approach requires that the researchers leading the work are committed to systematically involving all partners in the research process, and to recognizing and acknowledging the unique strengths that each partner brings. As such, researchers inform and give a voice to the community affected by the health condition and understand and value that this approach may reduce the autonomy and control of the research team (Fregonese, 2018; Tapp & Dulin, 2010). To achieve effective communication, scientists need to be willing to adapt technical language for the benefit of community leaders and advisory boards who are not familiar with scientific dialects (Fregonese, 2018). They also need to become familiar with methodologies such as focus groups, photo-voice, social network analysis, and ethnographic work (Kanamori et al., 2021a). Specific to genomics and neuroimaging research, this means explaining the methods, the data to be collected, and the ways the data will be used so that community members can easily understand. Models of successful science education efforts include public resources developed by the National Human Genome Research Institute (https://www.genome.gov/about-genomics), audiovisual media science communication disseminated by the Collaborative Studies on the Genetics of Alcoholism (COGA) project (https://cogastudy.org/aud/genes-in-aud/), Brain Awareness Campaign events sponsored by the Society for Neuroscience (https://www.sfn.org/outreach/brain-awareness-campaign), and coursework and activities geared towards high school students on the topics of genetics (https://cadrek12.org/projects/reducing-racially-biased-beliefs-fostering-complex-understanding-human-genetics-research) and neuroscience (Flanagan-Cato, 2019). However, more science education efforts are still needed to engage marginalized communities, such as increasing accessibility for non-English speakers (e.g., Budd et al., 2022) and disseminating these materials more broadly.

**Fig. 2:**
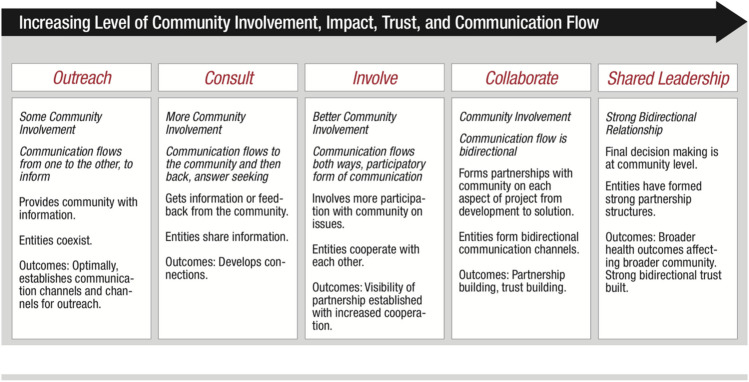
Researcher-Community Collaboration Continuum. Source: Clinical and Translational Science Awards Consortium. (2011). Principles of community engagement. Rockville, MD: US Gov. Printing Office

It is also important to display cultural humility in the creation of mutually respectful, equal, and dynamic partnerships between academic and underrepresented communities (Wallerstein & Duran, 2006). In other words, to create inclusive research approaches when integrating prevention science and biological science, we need to move the university-driven research agenda towards a mutually defined agenda or even a community-driven agenda (Wallerstein & Duran, 2006). Social network analysis can be used to identify and build a collaborative network of community partners (Kanamori et al., 2021b). Within CBPR approaches, the degree to which researchers and community partners collaborate can fall on a continuum, as illustrated in Fig. 2 (Clinical & Translational Science Awards Consortium, 2011). On the far right of the continuum, the goal is a truly equal partnership between scientists and underrepresented communities, where community members play a central role in decision-making, agenda-setting, and evaluating the appropriateness and priorities of future studies (Fregonese, 2018). By incorporating CBPR or aspects of CBPR approaches, prevention science-biomarker research has greater potential to improve an entire community’s health and reduce health disparities (Minkler & Wallerstein, 2003). As such, the unit of analysis expands from focusing on the health of a participant to the health of the community at large.

#### Establish a Diverse and inclusive Research Team

Establishing an inclusive and diverse workforce (clinicians, translational researchers, and basic science investigators) is a second way to responsibly integrate biological and prevention science (Clark & Hurd, 2020). As noted above, this is also an element of CBPR. Increasing the number of scientists from marginalized communities who are involved in integrated biomarker-prevention science research can increase the number of participants from marginalized communities in this research. Pipeline strategies that increase the number of early career researchers from underrepresented and marginalized groups, who have good qualifications in CBPR, but face challenges when submitting NIH applications as principal investigator because of current considerations as to what constitutes an excellent score for an application (Wallerstein & Duran, 2006), would facilitate this goal. Consistent with the CBPR approach discussed above, inclusive representation in prevention science-biomarker collaborations from marginalized communities is promoted when efforts to improve diversity in researcher leadership, including equal recruitment, retention, and promotion rates with respect to age, sex, gender, race, and ethnicity, are enacted. This type of paradigm shift requires changes in the current funding and academic performance evaluation systems (Fregonese, 2018). For example, there are currently disincentives to incorporating CBPR into biomarker-prevention science projects because of long development times to form true and sustainable partnerships, implement interventions collaboratively, and publish together with community members (Wallerstein & Duran, 2006). Promotion and tenure performance review committees would need to consider the time and efforts involved in implementing a study that incorporated CBPR approaches, due to the impact of the timing of data collection and publications and the inclusion of a combination of peer-reviewed scientific and non-peer-reviewed community-oriented publications (Fregonese, 2018). Early career faculty from marginalized communities also require protected time free from heavy administrative responsibilities, and benefit from mentoring by researchers who have required expertise (Wallerstein & Duran, 2006). When these strategies are incorporated at a systems level, a more diverse scientific field will develop, which will lead to more innovation and collective creativity.

#### Ensure Robust Collaborations Between Prevention Scientists and Biomarker Experts

Another important CBPR-based element for advancing research that integrates prevention science and biological science is to develop and nurture collaborative partnerships across disciplines. Similar to community partnerships, successful prevention scientist-biological scientist partnerships take years to establish. To earn respect and trust across disciplines, the team members need to establish a common language; understand the respective discipline-specific theories, methods, and analytic approaches; and have an agreed upon approach to collaboration and “who does what.” Common within-discipline activities such as writing a manuscript for publication are complicated when the work is multidisciplinary, as journal outlets, formats, lengths, and even authorship conventions may differ.

Once an effective partnership has been established, the team members can begin to discuss potential biological mechanisms and associated biomarkers that may be relevant, impacted, or invoked when a specific intervention is applied. It can be tempting (and certainly easier) to conduct a prevention trial and then measure a wide range of biomarkers to see what may have changed as a result of the intervention (see Fig. 1, Prevention Research Cycle, phase #2). However, atheoretical approaches are subject to Type I error, and do less to advance the field and the progression to the prevention research cycle phase #3 and beyond. Ideally, the team co-develops a theoretically grounded model of how a specific biomarker or system is part of a specific predictive pathway to a specific emotional or behavioral health outcome of interest before the work has been launched (prevention research cycle phases #1–2). Basic and applied research on transdiagnostic targets may be a good example—targeting these and learning about their biological correlates may be more effective than sticking to current diagnostic or preventative targets.

As noted earlier in Fig. 1, successful collaborations can create new knowledge that informs the prevention research cycle in bi-directional ways. For example, collaborative research in prevention research cycle phase #1 may indicate that parental scaffolding plays an etiological protective role on behavioral measures of child executive functioning. This might inform the development of a new study in the prevention research cycle phase #2, to add neuroimaging and a longitudinal element to examine the role of parental scaffolding on a biomarker of child executive functioning over time (e.g., prefrontal brain activity). If associations are identified, the research team may conceptualize an intervention to foster parental scaffolding, and measure whether changes in children’s executive functioning were identified as a result. This could be tested in a prevention research cycle phase #3 efficacy trial. The knowledge gained from this phase #3 trial may prompt the research team to theorize that *parental* executive functioning may also have an etiological role on associations between parental scaffolding and child executive functioning. A prevention research cycle phase #1 basic science study could then be initiated with a new sample, to test a modified theoretical model that tests a biomarker of parental executive functioning as a moderator of the association between parental scaffolding and child executive functioning (which could, in turn, inform future prevention targets in prevention research cycle phases #2–3).

#### Prioritize Improved Analytic and Multidimensional Modeling

There are a number of analytic and computational steps that can be implemented to help better integrate biological methods into prevention science research and interventions. The first pertains to how we model biomarker data. Historically, structural and functional neuroimaging studies examined brain regions in isolation of each other. There is growing recognition, however, that mental health and illness may be driven more by connections among brain regions than focal brain pathology (Basset et al., 2018; Braun et al., 2018; Menon, 2011). The emerging field of network neuroscience builds on a branch of mathematics called graph theory to model the connections between hundreds to thousands of regions of interests across the cortex and subcortex (Bassett et al., 2018). Similar developments are happening in bioinformatics and computational genomics to better understand the regulatory influence that genes have on each other and the principles of how DNA directs biology and molecular signaling pathways (Civelek & Lusis, 2014; Wei et al., 2014). These data-driven methods may enhance our mechanistic understanding of emotional and behavioral health and illness and provide new targets for both behavioral and pharmacological prevention and intervention efforts.

Next, most research on emotional and behavioral health and illness focuses on group-based statistics, examining how diverse groups differ on some outcome variable, or how one treatment compares to another. But group comparisons do not capture the heterogeneity of biological and psychological characteristics across any given outcome (Etkin et al., 2013; Insel & Cuthbert, 2015). Thus, any integrative approach to prevention science will ultimately need to model the individual at both biological and psychological levels of analysis. An example of this at the biomarker level is the recent development of “precision fMRI” approaches that use extended data acquisition and forward-thinking analyses of the functional connections in the brain to provide reliable and stable individual measures of brain organization (Gordon et al., 2017; Gratton et al., 2019). Early reports indicate that precision fMRI is more sensitive to individual differences and clinical symptoms than standard group-based analyses, and can increase the association between fMRI measures and behavior (Finn et al., 2015; Gordon et al., 2018; Kong et al., 2019). Future research is needed to examine whether precision fMRI techniques generate more individualized prognostic and diagnostic biomarkers, and more personalized targets in the brain for therapeutic interventions such as neuromodulation (e.g., transcranial magnetic stimulation, ultrasound). Paralleling precision fMRI is the development of personalized approaches to emotional and behavioral health that model variation in psychological symptoms and subjective experiences at the individual, rather than group, level of analysis (Wright & Woods, 2020). This development has been aided by advancements in the collection and sampling of longitudinal data (e.g., ambulatory assessment) and new statistical techniques that model dynamic processes of each individual’s psychopathology. An important direction for future research will be to integrate person-specific approaches to measuring brain activity (e.g., precision fMRI) with personalized models of emotional and behavioral health into a prevention science framework.

Finally, there are well-known concerns about the validity of the two major classification systems for psychiatric disorders currently in use (the ICD and DSM; Etkin et al., 2013; Krueger et al., 2018; Wright & Woods, 2020). These systems are not grounded in current psychological science, neuroscience, or genetics and do not appear to capture the fundamental mechanisms underlying emotional and behavioral health symptoms. This disconnect between diagnostic nosology and biological processes and mechanisms has most certainly contributed to the challenges in integrating biological and prevention sciences. Moving forward, it will be important for prevention science to align itself with forward-thinking and data-driven analytic methods for classifying psychiatric symptoms, including the NIMH’s Research Domain Criteria (RDoC; Insel & Cuthbert, 2015) and the Hierarchical Taxonomy of Psychopathology (HiTOP; Kotov et al., 2017).

#### Engage Research Teams in Conversations About Racism, Health Disparities, Language, and Ethical Issues

Given the long history of racism and other structural inequalities that have harmed science, harmed marginalized communities, and impeded progress in our ability to better integrate biomarkers into prevention science, we recommend that researchers who engage in biomarker-prevention science research also embed conversations and professional development activities about these topics into their research activities. Clark and Hurd (2020) recommend the inclusion of more proactive race-conscious or antiracism approaches to provide: (1) cognitive skillsets needed to identify and critically analyze biased assumptions, and (2) psychological tools required for healthy conversations about bias, racism, structural inequalities, and other social conditions that are perpetuating health disparities in the U.S. Further, we need increased awareness of the language we use in describing participants in biomarker research, with a recent National Academies of Sciences report (2023) recommending that researchers tailor their use of population descriptors based on the type and purpose of their study and explain why and how those descriptors were selected in their work. Their report offers a decision tree to help researchers choose whether race, ethnicity or indigeneity, geography, genetic ancestry, or genetic similarity are most appropriate for the work. By embedding these conversations, trainings, language, and commitment to learning from the field’s history and negative impacts (Gordon-Achebe et al., 2019), both established and early career prevention scientists will be better positioned to embark on prevention science-biomarker science with greater humility and respect for all persons, with the goal of improving the well-being of marginalized communities.

#### Include Relevant Expertise on Grant Review Panels and Journal Editorial Boards

Increasing the quality and quantity of integrated prevention science-biological science research requires that rigorous studies in this area are conducted and shared with scientific and community audiences. Possible solutions to accelerating the pace of translational research includes administrative actions such as ensuring that biologically oriented study sections and journal editorial boards include reviewers with prevention science and CBPR expertise, and vice versa. Similarly, inviting reviewers with interdisciplinary, integrative expertise may help provide relevant expertise to both promote research that is grounded in some of the principles laid out in this report, while also providing constructive critiques on research that may be lacking in one or more core ethical translational priority, to help guide future directions. Without scientists who are well versed across the spectrum—with training in working with marginalized communities and expertise in biological and prevention sciences, challenges will remain for this work to move forward, both because there will not be experts to lead it and because reviewers of papers and grants may not have the requisite expertise to review and appreciate the public health value of such work.

## Benefits and Challenges to Integrating Biomarker Science into Prevention Science Research Within a CBPR Context

Building on the strategies to promote the responsible integration of biological and prevention sciences described in the prior section, Table 1 presents an adaptation of the work of Hartwig and colleagues (2006) to describe a set of community benefits, research benefits, and challenges to consider when embarking on research that includes the integration of prevention science and biomarker science. As shown in Table 1, integrated prevention-biomarker collaborative research involves a series of steps, beginning with the assembly of a team of collaborators and progressing sequentially to activities such as defining the research questions and designing the project, conducting the study and intervention, analyzing and interpreting the data, and disseminating the findings. There are both community benefits and research benefits when researchers and the community work together in a collaborative prevention-biomarker study. Yet, there are also challenges in this type of collaborative research. In designing new collaborative studies in prevention-biomarker science, it is helpful to understand the unique benefits and challenges for the specific project early in the collaborative process. Unexpected challenges will likely still arise, but explicit conversations about benefits and challenges can help the team weather such challenges successfully. Table 1 highlights some of the common community benefits, research benefits, and challenges in prevention-biomarker collaborative science. The Table is intended to provide a high-level guide for researchers who wish to engage in integrated prevention science-biomarker research in the context of CBPR principles and approaches. The specific benefits to the community and researchers will necessarily need to be customized to the specific research topic and focal population. However, the challenges described in Table 1 are intended to serve as a guide for the team to consider and customize before embarking on a new project, to ensure the viability of a collaborative endeavor well in advance of asking for significant time, resources, and investment from the community.

**Table 1:** Proposed steps to apply a CBPR framework to integrated prevention-biomarker research: benefits and considerations

**Assemble a team of collaborators**	
Community benefits	• Community and research resources are used efficiently • Community members feel empowered • Representation is also prioritized when forming the research team • Community members may enjoy interacting with an interdisciplinary team
Research benefits	• Better probability of completing the project as planned • Diverse perspectives could generate important and unanticipated new questions
Challenges	• Takes time to identify the right collaborators with expertise in prevention science and/or the specific biomarker(s) of interest • Takes time to convince potential collaborators that they will play an important role in the project • Collaborators without community engagement experience may be less interested in or skilled at engaging in this type of integrated work
**Develop the structure for collaboration to guide decision making**	
Community benefits	• Trust is built (over time) • All members understand and accept human subject protection procedures
Research benefits	• Each collaborator shares their agenda • Clear roles and responsibilities for all partners in the research can improve teamwork and ultimately enhance the research through consideration of diverse perspectives
Challenges	• Takes time to build skills in group facilitation, consensus building, and group negotiation • Researchers who have not engaged with the community may have trouble sharing decision making or may not understand the value
**Define the research question**	
Community benefits	• Problems addressed are highly relevant to the community • Community members may enjoy learning about various research approaches (e.g., neuroimaging, genetics)
Research benefits	• Participants are motivated to invest their time in the project because it is viewed as relevant to them/their community • Research questions tailored to the community may be more acceptable to participants
Challenges	• Time consuming, yet sometimes decisions may need to be made with a rapid turn-around • The community may identify different issues than those identified by researchers, or for which funding is available • Community members may not perceive the relevance of measuring biomarkers or may have ethical concerns
**Design the project at a high level**	
Community benefits	• The community gains health knowledge and learns program design
Research benefits	• The community supports the research process • The community encourages members to participate • Designs that will be less appealing to participants are discarded
Challenges	• Study design may be more expensive and may take longer to implement • Possible threats to scientific rigor • Community may not have interest in some components of the study (e.g., biomarkers)
**Seek funding**	
Community benefits	• Aims of the grant proposal address issues that are important to the community • Community may gain knowledge of how to seek funding or learn of new funding sources
Research benefits	• Including community members on a steering committee or as co-investigators increases the likelihood of the application being funded • Additional funding opportunities may be available given the community partnership
Challenges	• Seeking input from the community slows the process and may complicate the proposal development, and sometimes funding opportunities have a very short turn-around timeline • Researcher’s goals may not align with community goals
**Recruit and retain participants**	
Community benefits	• Data collection approaches are acceptable to participants
Research benefits	• Participant recruitment and retention is easier and more effective • Participants are more motivated to be part of the project
Challenges	• Recruitment and retention approaches may be more complex, expensive and time consuming • The original data collection procedures may need to be modified • Larger samples sizes or different recruitment regions or sources may be required • Participants may be hesitant to provide biological samples • Informed consent documents may be more complicated to draft and review with participants
**Select study measures**	
Community benefits	• Measurement instruments are less likely to be offensive or biased • Measurement instruments are less likely to be confusing or misunderstood by participants
Research benefits	• Measurement instruments may have better reliability and validity for the population being studied • Less missing data if participants view the questions are acceptable, understandable, and appropriate
Challenges	• May be time consuming, particularly if cultural and/or linguistic adaptations or translations/back-translations are incorporated, and measurement invariance testing done • Possible threats to scientific rigor • May be less comparable to other studies if measures were modified or new measures developed for the study • Changes in the specific biomarker(s) collected and/or the collection methods may make them more acceptable, but with less interpretation power • Accommodating community members’ requests for modifications to measures or to biomarker collection protocols may make these less comparable to other studies or limit their interpretability
**Design and implement the intervention components**	
Community benefits	• Community feels the intervention is designed for and by them and offers benefits • Intervention provides resources to the community
Research benefits	• Increased likelihood of having the focal population feel positive about the study • Increased potential for sustainability beyond the initial study
Challenges	• Time consuming process of working together • Hiring community members may be less efficient than hiring staff • May take time to train community members • Universities may have barriers and/or delays in hiring community members, who may have extensive and relevant lived experiences but lack a higher education degree
**Analyze and interpret the data**	
Community benefits	• Community feels conclusions are accurate and sensitive
Research benefits	• Community supports the conclusions • Researcher less likely to be criticized for limited insight or cultural insensitivity
Challenges	• Interpretation of data by community may differ from that of researchers, calling for negotiation • Biomarker data is often so large/complex, may be difficult to negotiate or co-interpret with the community • Challenges identifying at what point in the analysis process community members should be involved
**Disseminate findings**	
Community benefits	• Community is proud of project accomplishments • Community gains experience in scientific writing that could facilitate career advancement • Findings are disseminated through outlets other than academic journals, making the science more accessible • Increased potential for project sustainability
Research benefits	• Findings are a more accurate reflection of the experiences of the community
Challenges	• Time consuming; requires extra mutual learning and negotiation • Community may disagree with how biomarkers are interpreted and what it should mean for translation • Challenges if study results indicate less positive outcomes for marginalized communities or people with marginalized identities

Note. Adapted from *Unit 1: Community-Based Participatory Research: Getting Grounded,* by K. Hartwig, D. Calleson, and M. Williams. (2006). In: The Examining Community-Institutional Partnerships for Prevention Research Group (Eds.), *Developing and sustaining community-based participatory research partnerships: A skill-building curriculum.* www.cbprcurriculum.info. Adapted with permission

### Examples of Studies That Have Infused CBPR Components into an Integrated Prevention-Biomarker Study

We have discussed the challenges in integrating prevention-biological science in research and provided some ready-to-implement strategies and frameworks that could move integration forward. In this section, we provide examples of prevention science research that have incorporated at least some of the strategies recommended throughout this article and presented in Table 1. Our examples are not comprehensive, rather, the purpose is to present a few studies that reflect different phases of the prevention research cycle and incorporate biomarker science, with consideration of at least one CBPR value or approach.

We draw specifically from prevention research cycle phases #1–3 (see Fig. 1). Knowledge from phase #1 can provide insights into biological mechanisms in the etiology of a behavior, such as stress response systems, that may be suitable for incorporation into phase #2 research to help identify new targets for prevention or intervention. Further, knowledge from phases #1–2 may lead to insights about specific mechanisms of change that could be incorporated into screening criteria in an efficacy study in phase #3, or, could be used to help select a subset of individuals who may be most likely to benefit from a particular intervention in a phase #3 study. Specifically, scores or thresholds of a biological measure may be reliable ways to discern for whom a particular psychosocial intervention may be most effective, using moderation analyses. Biomarkers can also be incorporated into phase #2 to measure the ability of a preventive intervention to serve as a mechanism of change (mediational analysis), and into phase #3 to measure efficacy of an intervention on behavioral or cognitive outcomes, as well as biomarkers. Prevention research phases #4 and #5 involve large-scale community trials and rollout of effective programs. We were unable to locate relevant examples of integrated biomarker-prevention science research that mapped directly onto phases #4–5. Given the additional challenges that are present when integrating biomarkers into the latter phases of the prevention research cycle and the current state of the science, we recommend that human health advances are most likely to occur in prevention research cycle phases #1–2, with application to phase #3 efficacy trials to test the processes and mechanisms identified in earlier stages using prospective designs, within a tightly controlled research study.

#### Prevention Research Cycle Phase #1: Basic Science Research that Identifies Risk and Protective Factors

As noted in Fig. 1, prevention research cycle phase #1 consists of basic science research that can provide information about biological and environmental risk and protective factors in the etiology of a behavior, that may then be suitable for subsequent incorporation into a prevention research cycle phase #2 or #3 study. One example of research in phase #1 is research from the ABCD study that examined associations between income, brain structure, and mental health, while considering how state-level policies such as anti-poverty programs may impact these associations (Weissman et al., 2023). There is a growing body of work examining the neuroscience of socioeconomic status and proposing that the brain is an entry point or pathway through which poverty and adversity become embedded in biology to generate these disparities (Hyde et al., 2020; Nusslock & Farah, 2022). To address this question, over 10,000 9- to 11-year-old youth from 17 states participated in a neuroimaging assessment, and associations with family income and youth psychopathology were examined (Weissman et al., 2023). Lower family income was associated with smaller hippocampal volume, and this association was stronger in states with a higher cost of living. However, the authors also identified a benefit of policies in some states that provided more income for low-income families (e.g., those that provided more cash benefits via Earned Income Tax Credits and Temporary Assistance for Needy Families). In such instances, the socioeconomic disparities in hippocampal volume were reduced by 34%, such that the association of family income with hippocampal volume in states with more generous benefits resembled that in the lowest cost of living states (with a similar pattern for child depression as an outcome). This study provides one example of how anti-poverty state-wide policies could impact associations between family income and a biomarker (hippocampal volume), in some settings. Also, see work from the Baby’s First Years study suggesting that monthly unconditional cash transfers to low-income families may have an impact on infant brain activity (Troller-Renfree et al., 2022). Prevention science researchers interested in examining mechanisms of change related to cash assistance programs and policies in phase #2 or #3 prevention research studies may benefit from including neuroimaging, if they hypothesize biological impacts in specific brain regions of a specific policy or practice.

#### Prevention Research Cycle Phase #2: Biological Mechanisms of Change Identified via a Prevention Study (Biomarkers as Mediators)

There are several examples from the field of prevention science that document intervention-related changes in a hypothesized biological mechanism. The Bucharest Early Intervention Project (BEIP) serves as one example by leveraging neuroimaging methods to elucidate the effects of psychosocial deprivation on brain development and cognitive functioning. In this study, researchers examined the development of infants and young children residing in institutional care who were randomly assigned to either a high-quality foster care or to care as usual (typically prolonged institutional care; Zeanah et al., 2003). This randomized controlled trial required navigating the complex ethics of conducting rigorous prevention science with vulnerable populations (Zeanah et al., 2012). The experimental design affords greater confidence in examining causal pathways from psychosocial deprivation to a host of negative developmental sequelae thought to be mediated through altered brain development. Structural magnetic resonance imaging (MRI) was initiated at 8 years and additional MRI assessments were conducted at 16 years. Study results indicated that foster care was an effective intervention in mitigating reduced cortical white matter volume associated with early deprivation (Sheridan et al., 2012). Moreover, specific white matter tracts contributed to these improvements, such as those involved in limbic and frontostriatal circuitry (Bick et al., 2015). Longitudinal examinations have shown greater cortical thinning from middle childhood to adolescence for children originally randomized into foster care compared to institutionally reared children, mirroring normative patterns of neural restructuring that occur across this development transition. Taken together, the BEIP studies highlight how specific biomarkers identified via neuroimaging may serve as mechanisms of action of the intervention “under the skin.”

A second example comes from the Strong African American Families study, a family skills training program aimed at mitigating the negative effects of poverty and life stress on rural African American youths through a focus on youths, parents, and their family interactions (Brody, 2016). As young adults (approximately age 25 years old), the same individuals who participated in the original intervention completed fMRI scans. Increased connectivity between the hippocampus and ventromedial prefrontal cortex was noted in the intervention group compared to controls—suggesting a mechanism of action of the adolescent intervention on brain connectivity in young adulthood (Hanson et al., 2019). Furthermore, individual gains in self-regulation, instilled by the intervention, statistically explained this brain difference. These results begin to connect neurobiological and psychosocial markers of risk and resiliency. The Strong African American Families and the BEIP examples both identified a biomarker indicative of a possible mechanism of change appearing years after the original intervention. A new study that proposed to examine these hypothesized biomarkers before and after the intervention in an efficacy trial would represent the progression of this work to the prevention research cycle phase #3.

#### Prevention Research Cycle Phase #2: Examining Whether Intervention Efficacy Is Predicated on a Biological Variable (Biomarkers as Moderators)

Perhaps the area with the most examples of integrated prevention science-biomarker research falls within this area of prevention research cycle phase #2, where researchers have examined whether the effects of an intervention (either a psychosocial intervention or a policy-level intervention) vary as a function of a specific genetic biomarker. Some of the advantages to this line of research are that: (1) one’s inherited DNA sequence does not change, and thus, retroactive collection of DNA in established prevention programs can be a relatively easy way to examine genetic associations across development, regardless of when DNA collection was initiated; (2) retroactive collection allows for participant-investigator rapport to be firmly established, engendering a trust that can facilitate collection of biological data, as described in an earlier section of this manuscript focused on CBPR methods; (3) random assignment to intervention eliminates person-level selection and the confound of gene-environment correlation; (4) random assignment increases statistical power, optimizing the detection of gene-environment interactions; (5) intervention designs are often longitudinal, enabling tests of distal intervention effects.

The Project Alliance 1 (PAL1) and Early Steps Multisite (ESM) studies, both large, randomized control trials of the Family Check-Up (FCU) intervention (at different developmental periods), are examples of prevention research cycle phase #2 studies that examined whether the intervention’s effects differed based on one’s genetics. The FCU is a brief psychosocial intervention designed to reduce youth problem behaviors by enhancing family management practices (Dishion & Stormshak, 2007; Dishion et al., 2008; Gill et al., 2008). Both PAL1 and ESM samples are racially/ethnically diverse, with the latter leveraging multi-site recruitment to maximize diversity. Both studies collected DNA well after launch, when participants were 27 and 14 years old, respectively. Using a “gene-by-intervention” analysis approach, each study documented intervention effects that varied as a function of participants’ genetic variation. Specifically, the FCU’s effects on maladaptive conduct problem trajectories, peer rejection, and substance use problems interacted with an individual’s polygenic score that indexed genetic risk for aggression and alcohol dependence (e.g., the intervention attenuated the link between relevant genetic risk and maladaptive outcomes; Elam et al., 2021, 2022; Kuo et al., 2019; Shaw et al., 2019). Moreover, children with greater genetic propensity towards environmental sensitivity showed a greater decrease in internalizing symptoms compared to those assigned to the control group, meaning that this polygenic score may have helped to identify youth who were most receptive to the positive effects of the intervention in preventing internalizing symptoms (Lemery-Chalfant et al., 2018). These emerging findings highlight the promise of genetically informed prevention science. As new and more highly powered GWAS are published and made available, prevention scientists can compute new polygenic scores and test associations with diverse phenotypes a priori, in prevention research cycle phase #2 or #3 studies. Creating a dynamic bank of polygenic scores illustrates another important advantage of integrating genetics into established prevention programs—the ability to generate new variables (assuming proper consent was obtained) without incurring further participant burden. Moreover, this science is evolving rapidly and novel genetic methods that stand to further enhance prevention science are on the horizon. For example, the Joint (Epi)genetics of Parenting and Stress Reactivity in the Development of Youths (JEOPARDY) study will implement a randomized control trial of the FCU and examine intervention effects on gene expression (Overbeek et al., 2020).

### Ongoing Barriers, Future Directions, and Recommendations for Researchers

Biological sciences have made significant advances over the past two decades, making technologies such as genomics and neuroimaging increasingly accessible to researchers. This has led to an increase in the uptake of biomarkers into prevention science studies, with multiple examples across prevention research cycle phases #1–2 completed and many more studies currently underway. *Prevention Science* has published a modest number of studies that integrate biomarkers in the last decade, most of which focus on genetics and were included as part of the 2018 special issue, ‘Incorporating Genetics in Prevention Science: Considering Methodology and Implications.’ Providing channels like this for the publication of burgeoning prevention-biomarker science will be key in advancing this work. Despite this progress, the field of integrated prevention-biomarker science is still quite young, and due both to the relative recency of the field as well as an unanticipated complexity of the science, there are challenges that the field needs to overcome in order to advance an equitable approach to integrated prevention science-biomarker science research. Some of the more prominent challenges discussed in this report include the lack of diversity in participants and researchers who are involved in the research; a lack of community engagement in all stages of the research; data and measurement issues such as small samples and/or small effect sizes, measurement reliability and validity issues; and ethical considerations. Given these challenges and the core value of prevention science of improving the lives of marginalized communities and people with marginalized identities, we recommended more comprehensive integration of CBPR approaches into research aimed at integrating prevention science with biomarker science. In cases where the relevant biomarker data and prevention science-relevant data have already been collected, it is not too late to consider basing the investigation in CBPR principles using some of the approaches discussed in this report.

Ongoing barriers and questions remain that have not been specifically discussed in this report, as we focused primarily on prevention research cycle phases #1–2. But as the work and methods advance to later stages of the prevention research cycle, research teams will need to consider the relevance of this work to policy makers, how to ethically implement large-scale biomarker collections in community settings in the context of effectiveness studies, and whether the investment of time and resources are best spent in biomarker collections or in providing additional direct services to the focal community. Further, it is anticipated that there will be ongoing advances in specific biomarker approaches and methods, and research teams will need to prioritize continued partnerships and training to maximize the likelihood that they will maintain the requisite expertise in the specific biomarker methods. As part of this training and ethical responsibility, it is essential that prevention scientists train and provide opportunities for early career prevention scientists with marginalized identities to become the next leaders in integrated prevention science-biomarker science that is steeped in CBPR approaches.

In closing, engaging in an equitable approach to integrated prevention science-biomarker science can lead to both scientific and community benefits. To maximize these potential benefits and minimize unintended harms, we recommend that prevention science researchers self-reflect on a series of questions before embarking on such endeavors: (1) Is there a theoretical rationale for the inclusion of a biomarker?; (2) Does the team include experts in the specific biomarker science?; Does the team include experts from the community or focal population? (3) Has the study team thoroughly educated themselves about the historical and current context related to their research question and focal population?; (4) Has input and consultation from the community or focal population been collected in the design of the research study? If so, is there support from the community for the research? Can members of the community participate as part of the research process?; (5) If the study is successful and the hypotheses are supported, will the study provide new knowledge that has reasonable potential to directly or indirectly benefit the focal population?; and (6) Is there potential for the community to use the knowledge generated from the research to sustain or apply the work after the research study and any associated funding are concluded? In this self-reflection, if the researchers answer ‘no’ to any of these questions, we recommend that they pause and revisit their approach and/or research questions until approaches that are more likely to promote health equity can be developed.
